# Efficacy of repetitive transcranial magnetic stimulation for disorders of consciousness: a retrospective cohort study

**DOI:** 10.3389/fneur.2025.1581467

**Published:** 2025-09-25

**Authors:** Shiqiang Zhang, Shengdong Liu, Chencong Gu, Ruijuan Zhang, Yushan Zhang, Mingchen Wang, Xiaodong Xu

**Affiliations:** ^1^Hebei Province Key Laboratory of Integrated Traditional and Western Medicine in Neurological Rehabilitation, Cangzhou Hospital of Integrated Traditional Chinese and Western of Hebei Province, Cangzhou, China; ^2^Cangzhou Central Hospital, Cangzhou, China

**Keywords:** repetitive transcranial magnetic stimulation, disorders of consciousness, Glasgow coma scale, influencing factors, retrospective study

## Abstract

**Objective:**

To evaluate the efficacy and safety of repetitive transcranial magnetic stimulation (rTMS) in the treatment of patients with disorders of consciousness (DoC) in a real-world setting, and to analyze the relevant factors affecting efficacy.

**Method:**

Using a single-center retrospective cohort study design based on a hospital information system, we reviewed all patients with DoC presenting to the rehabilitation unit of our hospital between October 2019 and October 2024. Efficacy was assessed using the Glasgow Coma Scale (GCS), with the presence or absence of rTMS as an exposure factor.

**Results:**

The exposed group did not significantly improve the GCS scores of patients with DoC compared to the non-exposed group. The Subgroup analysis showed that rTMS improved the level of consciousness in patients with stroke compared to the non-exposed group (*p* < 0.05), but there was no statistical significance in the comparison between the groups of patients with traumatic brain injury. Binary logistic regression analyses showed that shorter disease duration, injury at non-brain stem sites, higher pretreatment GCS scores, earlier timing of intervention, and combined use of acupuncture, amantadine, piracetam, and Suhexiang Pill were independent factors influencing the good prognosis of DoC patients.

**Conclusion:**

rTMS did not significantly improve the GCS scores of patients with DoC. However, it may improve the level of consciousness of patients with stroke-induced or moderate DoC. Nevertheless, this conclusion requires validation through rigorous, standardized, large-sample randomized controlled trials (RCTs).

## 1 Introduction

Disorders of consciousness (DoC) is a clinical condition caused by severe brain injury, defined as damage to the superior reticular activating system and/or bilateral hemispheres, resulting in a reduction or loss of the organism’s ability to become aware of and perceive the environment (1–3). DoC can be caused by a variety of conditions. These include traumatic brain injury, stroke, hypoxic–ischemic encephalopathy, and tumor burden (4). The poor quality of life of patients with DoC causes a great deal of emotional distress to their families and an economic burden for society (5). In addition, the incidence of DoC is gradually increasing as the population ages and emergency care techniques improve (6). Therefore, the search for effective treatments to improve the level of consciousness in DoC patients is an important issue and a challenging topic in neuroscience.

However, there is a lack of clear and effective treatments for DoC. Large sample case studies in this area are still lacking. Patients with DoC currently rely mainly on long-term medication, rehabilitation therapy and rehabilitation care at this stage. Current treatment options for DoC are still largely empirical, with a lack of evidence-based medical research, and only amantadine and transcranial direct current stimulation are considered to have secondary evidence (7). A number of clinical treatments, including medications, non-invasive brain stimulation, invasive deep brain stimulation and spinal cord electrical stimulation surgery, offer hope for improving the condition of patients with DoC (8–10). The therapeutic interventions used in rehabilitation, such as postural transfers, exercise therapy, multisensory stimulation training, music therapy and other programs, have been observed in relevant studies to produce partial behavioral improvements or changes in neurological imaging, but no significant improvement in clinical scores (11, 12). In addition, factors such as different etiologies and disease severity also affect the prognosis of patients with DoC. Clinical and neuroimaging data are a challenge to the lack of choice in treatment selection (13).

A recent review points to non-invasive neuromodulation as a promising intervention, including repetitive transcranial magnetic stimulation (rTMS), but the therapeutic efficacy of rTMS is still inconsistent (14). rTMS is a non-invasive neuromodulation technique. In recent years, rTMS has received increasing attention in the treatment of DoC (15–17). However, the limited data available on rTMS is viewed with cautiously by most clinicians (18). rTMS improves brain function by generating a rapidly changing magnetic field that penetrates the skull and acts on the cerebral cortex to regulate neuronal activity (19). rTMS induces functional correlations between the default mode networks and the external perceptual networks (20). A randomized controlled trial of patients in a minimally conscious state (MCS) revealed that those who received 10-Hz rTMS showed greater improvement in level of consciousness, EEG activity, and disturbance complexity index than the group that received sham stimulation (21).

Repetitive transcranial magnetic stimulation has shown promise in the treatment of DoC. However, a number of questions and challenges remain (22, 23). For example, the sample sizes of the trials were small and there was heterogeneity in patients with different etiologies, disease duration, lesion sites and stimulation protocols. This makes it difficult to draw consistent conclusions about the efficacy of rTMS. The optimal stimulation parameters for rTMS therapy are not fully defined. There are individual differences in the response to rTMS in different patients, and some patients may experience adverse effects such as dizziness and scalp pain (24). A randomized controlled trial showed that real rTMS-treated DoC patients had a significant improvement in consciousness compared to sham rTMS stimulation. However, in-depth analysis showed that only some patients with active rTMS induction had a significant increase in awareness scores and that rTMS did not significantly improve arousal rates (25). This suggests that it is important to identify potential patients whose level of consciousness can be improved by rTMS.

There is limited and insufficient evidence for rTMS for DoC (26). Therefore, there is a need for a more comprehensive study of the efficacy and safety of rTMS in the treatment of patients with DoC, as well as an analysis of the factors that influence efficacy.

## 2 Research programs

### 2.1 Study design

**Figure 1:**
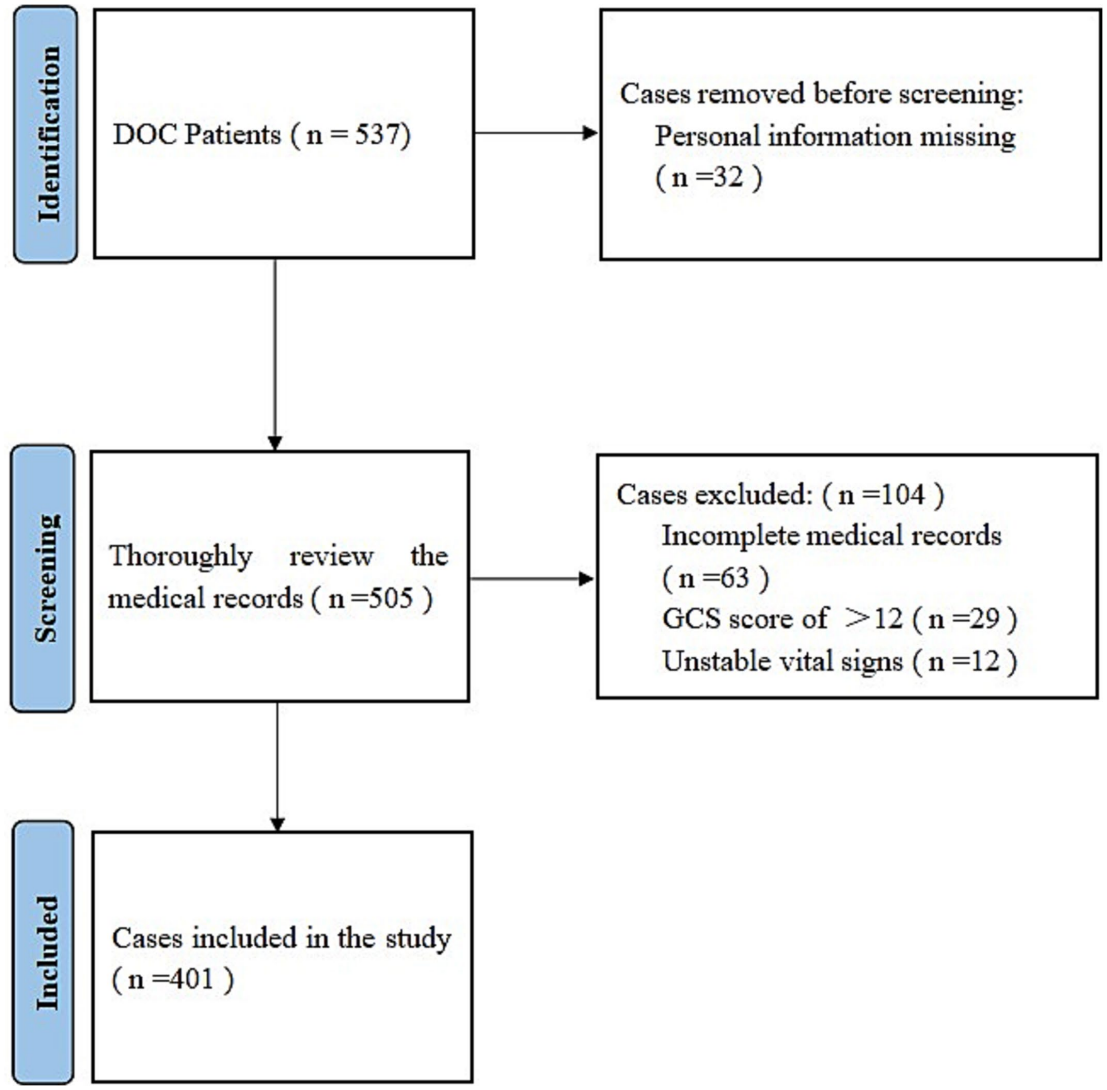
Flow diagram for enrolling participants.

The study design was a single center retrospective cohort study. The hospital information system was used, with DoC and Glasgow Coma Scale (GCS) as search terms. The search period was set from October 1, 2019 to October 1, 2024 for primary screening. The study was conducted in patients with DoC, with or without rTMS as an exposure factor, and with GCS to assess the efficacy of wakefulness promotion. The safety of the study was assessed by the presence or absence of aggravation of brain injury, seizures, dizziness and headache. The study protocol was approved by the Ethics Committee of the Cangzhou Hospital of Integrated Traditional Chinese and Western of Hebei Province (Approval number: CZX2023-KY-063.1). The study is a retrospective study of medical records and does not require patients to sign an informed consent form. The process of participant inclusion is illustrated in Figure 1. It was created using the PRISMA flowchart generator.

### 2.2 Data collection

All patients admitted to the rehabilitation unit of our hospital were included in the study. Collection of basic patient information, such as sex, age, etiology, comorbidities. Review of patients’ GCS scores on admission and discharge using the Zhoudao system. Collect information on adverse events documented in the medical record. Examples include induced seizures, exacerbation of brain injury, dizziness and headache. Collect medications such as amantadine, baclofen, cytarabine sodium, piracetam, xingnaojing injection, donepezil, suhagra pills. Collection of information on rehabilitation programs such as exercise therapy, swallowing therapy, transcranial direct current stimulation, hyperbaric oxygen therapy, music therapy, low-frequency electrical stimulation therapy, acupuncture.

### 2.3 Inclusion criteria

Fulfills the diagnosis of DoC and has a GCS score of 3–12. Patients with DoC due to various causes, including traumatic brain injury, stroke, ischemic and hypoxic encephalopathy, brain tumors, and others. No gender restrictions. No restriction on duration of illness. First episode of DoC. Complete medical record data collection.

### 2.4 Exclusion criteria

Patients with unstable vital signs. Patients with missing data from the medical record. Patients with severe skull defects that affect rTMS coil placement. Patients with rTMS contraindications, such as intracranial metal implants or a history of epilepsy.

### 2.5 Grouping

Classification into exposed and non-exposed groups based on their use of rTMS.

### 2.6 Intervention methods

Patients in both groups were routinely monitored for blood pressure, electrocardiogram and oxygen saturation. They also received medication to treat their symptoms. Targeted rehabilitation was provided according to the patient’s specific dysfunction, including passive activities, swallowing stimulation, etc. In addition, the exposure group was treated with rTMS. The stimulation parameters were as follows.

Stimulus location: Left Dorsolateral Prefrontal Cortex (DLPFC_L); Stimulus intensity: 90%; Increment time: 0 s; Stimulus frequency: 10 Hz; Span: 2.5 s, Interval time: 10 s; Number of repetitions: 52; Treatment time: 10min40s; Total impulses:1300.

Instrumentation: Brain Ultimate Transcranial Magnetic Stimulation M Series. Model code: BY90A. Factory: SHENZHEN YINGZHI TECHNOLOGY COLTD.

### 2.7 Observation indicator

#### 2.7.1 GCS

This is a widely used scale to measure the level of consciousness of people with brain injury and consists of three sections: eye opening response, verbal response and motor response (27). The total score ranges from 3 to 15 points. The higher the score, the higher the level of consciousness of the patient. According to the score, consciousness levels are divided as follows:

- 3–8 points: coma.
- 9–12 points: moderate DoC.
- 13–14 points: mild DoC.
- 15 points: clear consciousness.

### 2.8 Adverse reaction records

During and within half an hour after rTMS treatment, adverse reactions such as seizures, aggravation of brain damage, dizziness and headache were recorded.

### 2.9 Statistical methods

SPSS 26.0 software was used to process the data. Measures conforming to a normal distribution were tested using *t*-tests. They are expressed as mean ± standard deviation ( $\bar{x}\pm s$ ). Data with skewed distributions were tested using non-parametric tests and expressed as median (interquartile range). For subgroup analysis, we will use Bonferroni for multiple comparison correction. The chi-squared test was used for count data. If the baseline data of the two groups differed too greatly, a multivariate regression model was employed to account for confounding factors. Univariate analysis of factors associated with rTMS efficacy using chi-squared test. Significant factors were then analyzed using binary logistic regression analysis. *α* = 0.05 was chosen as the level of significance and *p* < 0.05 was considered statistically significant.

## 3 Results

### 3.1 Baseline characteristics

Comparisons between the two groups were not statistically significant for sex, age, disease duration, etiology, whether or not craniotomy was performed, lesion location, underlying disease, whether or not tracheotomy was performed, pulmonary infections, and increased muscle tone. Further details are shown in Table 1.

**Table 1:** Analysis of demographic characteristics of participants.

Variables	Categories	Non-exposed group (*n* = 152)	Exposed group (*n* = 249)	*p-*value
Gender	Male	109	178	0.961
	Female	43	71	
Age (years)	$\bar{x}\pm s$	58.00 (47.00,67.00)	57.00 (49.50,68.50)	0.868
Disease course (days)	$\bar{x}\pm s$	38.00 (28.00,70.00)	36.00 (27.00,87.00)	0.597
GCS scores	3–8 scores	121	182	0.141
	9–12 scores	31	67	
Cause of illness	Traumatic brain injury	43	76	0.564
	Stroke	102	158	
	Ischemic–hypoxic encephalopathy	6	9	
	Other	1	6	
Craniotomy	Yes	97	137	0.083
	No	55	112	
Location of lesion	Brain stem	63	106	0.825
	Non-brain stem	89	143	
Hypertensive	Yes	93	156	0.769
	No	59	93	
Diabetes	Yes	66	113	0.702
	No	86	136	
Coronary heart disease	Yes	47	81	0.901
	No	105	168	
Cerebrovascular disease	Yes	14	19	0.576
	No	138	230	
Tracheotomy	Yes	122	189	0.310
	No	30	60	
Pulmonary infection	Yes	121	180	0.100
	No	31	69	
Increase in muscle tone	Yes	71	140	0.064
	No	81	109	

GCS, Glasgow coma scale.

### 3.2 Comparison of GCS scores

Since the two sets of data do not conform to a normal distribution, a nonparametric test is used. Comparisons of GCS scores between the two groups were not statistically significant at either the pre- or post-treatment levels (*p* > 0.05). Further details are shown in Table 2. A multiple linear regression model was used to control for possible confounding factors. Even after controlling for disease course, etiology, craniotomy, lesion location, and pulmonary infection, the results showed no statistical significance between exposure factors and outcomes (*p* > 0.05). This indicates that the results are reliable.

**Table 2:** Comparison of GCS scores between two groups of participants.

Outcome Measure	Time points	Sample size(Ng/Eg)	Non-exposed group	Exposed group	*p*-value	Estimated difference (95% CI)
GCS	Baseline	152/249	7.00(5.00,8.00)	6.00(6.00,9.00)	0.165	0.000(0.000,1.000)
	After treatment	152/249	8.00(7.00,9.00)	9.00(6.00,9.00)	0.061	0.000(0.000,1.000)

CI, Confidence interval; Eg, Exposed group; GCS, Glasgow coma scale; Ng, Non-exposed group.

#### 3.2.1 Subgroup analysis by etiology and level of consciousness

The comparison of GCS scores between the two groups was not statistically significant in traumatic brain injury patients either before or after treatment. There was no statistically significant comparison between the two groups in stroke patients before treatment. GCS scores were higher in the exposed group than in the non-exposed group after treatment in stroke patients, and the difference between the two groups was statistically significant. In comatose patients, the comparison of GCS scores between the two groups was not statistically significant either before or after treatment. In patients with moderate DoC, pre-treatment comparisons between groups were not statistically significant, and post-treatment GCS scores were higher in the exposed group than in the unexposed group, with a statistically significant difference between groups. Further details are shown in Table 3.

**Table 3:** Subgroup analysis by etiology and level of consciousness.

Categories	Time points	Sample size(Ng/Eg)	Non-exposed group	Exposed group	*p-*value	Mean difference (95% CI)
Traumatic brain injury	Baseline	43/76	6.00(7.00,9.00)	6.00(6.00,9.00)	0.798	−0.086(−0.899, 0.727)
	After treatment	43/76	8.00(8.00,9.00)	8.00(6.00,9.00)	0.503	−0.286(−1.316, 0.743)
Stroke	Baseline	102/158	7.00(6.00,8.00)	7.00(5.00,8.00)	0.186	0.265(−0.215, 0.744)
	After treatment	102/158	8.00(6.00,8.00)	9.00(7.00,9.00)	0.009	0.446(0.202, 1.093)
3–8 points	Baseline	121/182	6.00(5.00,7.50)	6.00(6.00,7.00)	0.957	0.008(−0.329, 0.344)
	After treatment	121/182	7.50(6.00,9.00)	8.00(7.00,8.00)	0.449	0.175(−0.379, 0.729)
9–12 points	Baseline	31/67	9.00(9.00,10.00)	9.00(9.00,9.00)	0.153	−0.218(−0.487, 0.051)
	After treatment	31/67	9.00(9.00,11.00)	9.00(9.00,12.00)	0.044	0.477(0.300, 1.253)

Eg, Exposed group; Ng, Non-exposed group.

### 3.3 Analysis of factors influencing the efficacy of rTMS

The exposure group was divided into two groups of patients based on post-treatment GCS scores, with ≤8 representing the poor prognosis group and >8 representing the good prognosis group. Univariate analysis to screen for possible influences followed by multifactorial analysis.

#### 3.3.1 Univariate analysis

Univariate analysis showed that disease duration, lesion location, pretreatment GCS scores, frequency of interventions, duration of interventions, use of transcranial direct current stimulation, use of low-frequency electrical stimulation, use of acupuncture, use of amantadine, use of piracetam, use of xingnaojing injection, use of donepezil, and use of SuheXiang Pill were statistically different between the good and poor prognosis groups (*p* < 0.05). More details are shown in Table 4.

**Table 4:** Comparison of univariate analyses between good and poor prognosis groups.

Variables	Categories	Good prognosis group (*n* = 129)	Poor prognosis group (*n* = 120)	*p-*value
Gender	Male	95	83	0.434
	Female	34	37	
Age (years)	≤60	83	67	0.170
	>60	46	53	
Disease duration (days)	1–30	48	60	0.000
	31–60	46	15	
	61–90	21	9	
	>90	14	36	
Cause of illness	Traumatic brain injury	36	40	0.424
	Stroke	86	72	
	Ischemic–hypoxic encephalopathy	3	6	
	Other	4	2	
Craniotomy	Yes	70	67	0.804
	No	59	53	
Location of lesion	Brain stem	32	74	0.000
	Non-brain stem	97	46	
Pretreatment GCS score	3–8	63	119	0.000
	9–12	66	1	
Frequency of intervention	1–14	4	16	0.012
	15–30	40	34	
	≥31	85	70	
Time of intervention	1–30	50	22	0.000
	31–60	49	22	
	61–90	11	35	
	≥91	19	41	
Hypertensive	Yes	75	81	0.127
	No	54	39	
Diabetes	Yes	61	52	0.531
	No	68	68	
Coronary heart disease	Yes	44	37	0.581
	No	85	83	
Transcranial direct current stimulation	Yes	49	30	0.028
	No	80	90	
Hyperbaric oxygen therapy	Yes	60	60	0.582
	No	69	60	
Music therapy	Yes	77	58	0.072
	No	52	62	
Low frequency electrical stimulation	Yes	119	90	0.000
	No	10	30	
Exercise therapy	Yes	120	110	0.687
	No	9	10	
Swallowing therapy	Yes	109	99	0.671
	No	20	21	
Acupuncture	Yes	126	110	0.033
	No	3	10	
Amantadine	Yes	96	34	0.000
	No	33	86	
Baclofen	Yes	20	20	0.779
	No	109	100	
Citicoline Sodium	Yes	9	11	0.525
	No	120	109	
Piracetam	Yes	40	20	0.001
	No	89	100	
Xingnaojing Injection	Yes	67	42	0.007
	No	62	78	
Donepezil	Yes	66	4	0.000
	No	63	116	
SuheXiang Pill	Yes	92	38	0.000
	No	37	82	

GCS, Glasgow coma scale.

#### 3.3.2 Multifactorial analysis

Binary logistic regression analyses showed that shorter disease duration, injury at non-brainstem sites, higher pretreatment GCS scores, earlier timing of intervention, and combined use of acupuncture, amantadine, piracetam, and Suhexiang Pill were independent factors influencing the good prognosis of DoC patients. Further details are shown in Table 5.

**Table 5:** Binary logistic regression analysis.

Factors	*B*	SE	*Wald χ*^2^	*p-*value	Exp (B)	95% CI
Disease duration (days)			9.461	0.024		
1–30	2.691	1.756	2.348	0.125	14.753	0.472,461.264
31–60	4.164	1.710	5.934	0.015	64.360	2.257,183.637
61–90	3.356	1.257	7.125	0.008	28.661	2.439,336.783
Location of lesion (Non-brain stem)	1.094	0.496	4.871	0.027	2.987	1.130,7.896
Pre-treatment GCS score (9–12)	−7.726	1.376	31.548	0.000	0.000	0.000,0.007
Frequency of intervention			0.188	0.910		
1–14	0.089	1.055	0.007	0.933	1.093	0.138,8.640
15–30	−0.212	0.525	0.163	0.687	0.809	0.289,2.265
Time of intervention			18.404	0.000		
1–30	−4.889	1.937	6.371	0.012	0.008	0.000,0.335
31–60	−0.466	1.724	0.073	0.787	0.627	0.021,18.407
61–90	−1.693	1.298	1.700	0.192	0.184	0.0,14,2.344
Transcranial direct current stimulation (use)	−0.877	0.707	1.539	0.215	0.416	0.104,1.663
Low frequency electrical stimulation (use)	1.641	0.996	2.717	0.099	5.162	0.733,36.339
Acupuncture (use)	−6.893	2.605	7.003	0.008	0.001	0.000,0.167
Amantadine (use)	−1.691	0.352	23.142	0.000	0.184	0.093,0.367
Piracetam (use)	−0.936	0.449	4.335	0.037	0.392	0.163,0.947
Xingnaojing Injection (use)	−0.511	0.327	2.438	0.118	0.600	0.316,1.139
SuheXiang Pill (use)	−2.735	0.560	23.881	0.000	0.065	0.022,0.194

GCS, Glasgow coma scale.

### 3.4 Safety records

In this study, we found that one subject had a petit mal seizure during rTMS treatment, which lasted approximately 1 min and resolved spontaneously, and did not have another seizure during subsequent treatment. There were no reports of aggravation of brain damage, dizziness, or headache with rTMS during the course of the disease.

## 4 Discussion

This study carefully analyzed the effect of rTMS treatment on the level of consciousness in patients with DoC and the relevant factors influencing treatment efficacy using a large data set over 5 years. To the best of our knowledge, based on our review of the literature, this is the largest sample size rTMS study conducted to date in patients with DoC, providing insights for clinical decision-making and future research.

### 4.1 Efficacy of rTMS in the treatment of patients with DoC

In this study, we analyzed the degree of improvement in the level of consciousness of DoC patients treated with rTMS by retrospectively analyzing DoC patients admitted to the rehabilitation unit of our hospital between 2019 and 2024. We found that rTMS did not significantly improve the level of consciousness in DoC patients. This finding is contrary to the conclusion of some randomized controlled trials (15, 17, 19). The reason may be related to differences in the populations included in the studies. Previous positive studies mostly focused on the MCS following trauma. In contrast, this study included a wider range of causes, such as stroke, trauma, ischemia, and hypoxia. Different etiologies result in different patterns of neural network damage and plasticity potential. This dilutes the overall therapeutic effect. Therefore, further subgroup analyses were performed according to the cause of brain damage. Subgroup analyses of patients with ischemic–hypoxic encephalopathy and other causes of DoC were not performed due to the small number of patients included. A subgroup analysis of traumatic brain injury and stroke only showed that rTMS improved level of consciousness in patients with post-stroke DoC, but did not significantly improve level of consciousness in patients with traumatic brain injury. Consider that the difference in outcome may be related to the following factors: Traumatic brain injury usually involves extensive damage to brain tissue, including cerebral contusion and intracerebral hemorrhage, which result in more severe neuronal cell death and structural damage to brain tissue, and may induce extensive dysfunction of the brain’s neural network. rTMS promotes functional recovery mainly by modulating neuronal excitability and may have a limited role in repairing this structural damage. A study of the efficacy of rTMS in the primary motor cortex of patients in a vegetative state found no significant increase in their level of consciousness (28). There are similarities with the results of this study. After a stroke, especially an ischemic stroke, brain tissue shows neurological dysfunction mainly due to ischemic and hypoxia. rTMS may promote neuroplasticity by modulating the excitability of the cerebral cortex and help restore damaged neurological function, similar to the results of this study (29). However, rTMS has also been found to increase the level of consciousness in patients with traumatic brain injury, which differs from the results of the present study (30, 31). Through the analysis, it was found that one is a pilot study with a small sample (30), and the other adopts a different stimulus scheme from this study (31). Therefore, the stimulus site and parameters should be reconsidered to address the disturbance of consciousness caused by trauma. In conclusion, rTMS is more effective in the treatment of DoC due to stroke, mainly because it can improve neurological dysfunction by modulating neuronal excitability and promoting neuroplasticity. However, it has limited effects on repairing structural damage in traumatic brain injury and the results of related studies are mixed.

The level of consciousness can be graded according to the GCS score, with 3–8 being coma and 9–12 being moderate DoC (27). No participants with a score of 2 or less were identified during the initial screening of the study, while participants with a score of 13–14 had mild DoC and incomplete documentation of GCS scores in their medical records. Based on these two objective factors, we analyzed only coma participants with scores of 3–8 and moderate DoC participants with scores of 9–12. In patients with moderate DoC, but not in comatose patients, rTMS was found to improve the level of consciousness. The reason for this finding may be related to the following factors: comatose patients have more severe brain damage and the plasticity of the neural network is more limited, whereas moderate DoC patients do not have a complete loss of neural function at the site of brain damage and the plasticity of the neural network is relatively good. A randomized controlled pilot study using the same stimulation protocol as the present study found that the inclusion of rTMS significantly increased the level of consciousness in MCS patients, similar to the results of the present study (21).

### 4.2 Stimulation parameters of rTMS for DoC treatment

First, it is important to note that the stimulation protocol used in this study is fixed, since this parameter was set on the device when this transcranial magnetic stimulation therapy device was introduced by the hospital. Our rehabilitation therapists are also required to follow this program during treatment. However, due to the short time that rTMS has been used for DoC and the fact that the relevant studies tend to be case reports, formal treatment protocols have not yet been established.

Repetitive transcranial magnetic stimulation is based on the principle of electromagnetic induction and induces neuronal depolarization in the brain to achieve the effect of modulating cortical excitability (32). In the rTMS stimulation mode, low frequencies are inhibitory and reduce neuronal excitability, while high frequencies increase cortical excitability (33). In previous studies on the treatment of DoC, rTMS was mostly used at 10 or 20 Hz. The results of a meta-analysis showed that 20 Hz rTMS promoted an increased level of consciousness in patients with DoC, but 10 Hz rTMS did not induce significant changes, which is different from the results of the present study (26). The present study found that 10 Hz rTMS can still have a positive effect on the level of consciousness of some patients with DoC. Another randomized controlled trial found that 10 Hz rTMS increased the level of consciousness in patients with chronic DoC (34). There was also a 10 Hz rTMS treatment that significantly improved the level of consciousness in patients with DoC, especially in those with the lowest level of consciousness (35). However, there is a lack of studies comparing 10 Hz and 20 Hz rTMS interventions, so it is not possible to determine whether one frequency is more effective or not. Therefore, there is a need for future research into the optimal stimulation frequency.

In order to activate a larger area of the cortex and to better increase the level of consciousness, there have also been some advances in rTMS in terms of the site of stimulation. The site of stimulation is selected by shifting from the primary movement cortex (M1) to the DLPFC, followed by a parietal cortex. Currently, the DLPFC is the main region stimulated by rTMS (36). rTMS targeting the DLPFC promotes recovery of consciousness (37). A meta-analysis found that patients with DLPFC as the stimulation area for rTMS had the most significant improvement in their level of consciousness (38). In addition, M1 is also a target region for rTMS in DoC patients (39, 40). One study found that rTMS stimulation targeting M1 improved consciousness in patients with DoC (41). However, a small sample study found that rTMS treatment targeting M1 was not effective in treating DoC (28). The results of another study also did not provide sufficient evidence for the effect of rTMS treatment targeting the left M1 on DoC (42). Therefore, application to M1 may not be the most appropriate target region for rTMS treatment of DoC.

A randomized controlled trial showed that 10 Hz rTMS over the posterior parietal cortex significantly promoted recovery of consciousness in patients with DoC (43). A recent preliminary study has found that targeting parietal rTMS improves neurobehavioral functioning and promotes frontal lobe activity in patients with long-term DoC, providing a novel target for treatment (44).

### 4.3 Factors influencing the efficacy of rTMS in the treatment of DoC

The prognosis of DoC may be related to the etiology of the patient’s disease, the severity of its onset, the appropriateness and timeliness of early treatment, the timing of rehabilitation, and the choice of interventions.

#### 4.3.1 Course and prognosis of DoC

If DoC has been present for more than 1 year, the prognosis for recovery of consciousness is poorer, especially if early improvement in consciousness is not significant (45). This study found that patients with a disease duration of 31–90 days had a better prognosis than patients with a disease duration of more than 90 days at enrollment. However, there was no significant difference compared to a disease duration of 1–30 days. This finding may be related to the early instability and severity of the patient’s condition, or the small sample size of 1–30 day participants. However, some studies have found that even months after the onset of DoC, recovery of consciousness may be facilitated by prolonged rehabilitation therapy (46).

#### 4.3.2 Injury site and prognosis of DoC

This study found that patients with non-brain stem injuries had a better prognosis. This may be due to the fact that brainstem injury is one of the major causes of DoC. The prognosis for DoC is usually more complex and severe (47). The brainstem contains important nerve nuclei and conduction pathways that are key structures for maintaining alertness and vital signs. Brainstem injury can lead to severe DoC and even prolonged coma, especially if the injury involves the superior reticular activating system of the brainstem (48). A retrospective analysis of DoC after stroke found a significant association between brainstem injury and poor prognosis for recovery of consciousness, similar to the results of the present study (49). While the effect of non-brain stem injuries on the level of consciousness varies depending on the location and severity of the injury (50). If the damage is limited to certain lobes of the brain, it may cause cognitive, motor and sensory dysfunction, but the effect on consciousness is relatively minor and patients may gradually regain some function after rehabilitation therapy. Therefore, the management and prognostic evaluation of patients with DoC must take into account the site of injury, the mechanism, and the individual characteristics of the patient. In this study, we divided the injury site into only two major categories, but the site that caused the patient’s DoC can be divided into many smaller areas. It is hoped that prospective studies based on subgroups of injury sites can be conducted in the future to further analyze the relationship between injury sites and the prognosis of patients with DoC, in order to provide a reference for the management of patients with DoC.

#### 4.3.3 Pre-treatment GCS score and prognosis of DoC

The lower the patient’s GCS score on admission, the more severe the damage to brain tissue, the deeper the coma and the higher the risk of irreversible damage to nerve cells, indicating a poorer prognosis (51). This study found that patients with moderate DoC on admission had a better prognosis compared to comatose patients, and of course this result occurred in relation to the subgroups we defined for prognosis.

#### 4.3.4 Timing of intervention and prognosis of DoC

This study found that patients who underwent intervention on days 0–30 had a better prognosis than those who underwent intervention >90 days. Therefore, it is recommended that rTMS be administered to patients with DoC as early as possible after their condition has stabilized, as this may help to improve the patient’s prognosis.

#### 4.3.5 Acupuncture and prognosis of DoC

Acupuncture as a traditional Chinese medicine therapy shows a role in the rehabilitation of DoC patients. This study found that patients treated with combined acupuncture had a better prognosis. A multicenter cohort study analyzing the effect of acupuncture on the recovery of consciousness in patients with acute traumatic brain injury revealed that patients who received acupuncture experienced greater improvement in their GCS scores than those who did not. This finding is similar to the results of the present study (52). Another study, using functional near-infrared spectroscopy, found that acupuncture increased the concentration of oxygenated hemoglobin in the frontal cortex and improved the strength of connections in the left cerebral cortex, which had a positive effect on the prognosis of patients with DoC (53).

#### 4.3.6 Drugs and prognosis of DoC

Drugs are the conventional treatment of choice for DoC, but there is also a lack of consistent guideline recommendations for drug selection. Only amantadine was rated as a secondary recommendation (7). This study found that the combined use of amantadine, piracetam and Suhexiang Pill increased patients’ level of consciousness. Piracetam, a classic drug used to improve cerebral metabolism, has been shown to be effective in some people with DoC and may be beneficial in restoring consciousness by improving cerebral blood flow and having antioxidant effects (54, 55). Suhexiang Pill is widely used as a “wake-up” drug in Chinese clinics. Suhexiang Pill is a traditional Chinese medicine compounded preparation belonging to the category of orifice opening aromatic drugs, with the effect of opening the orifices and awakening the mind. The study found that Suhexiang Pill significantly improved patients’ level of consciousness and reduced the incidence of related complications (56). This is similar to the results of this study.

Although this study did not find an effect of other factors on the level of consciousness. However, in our clinical rehabilitation work, we have found that many factors, including age, comorbidities, nutritional support, and the timeliness and intensity of rehabilitation treatments, have an impact on patient prognosis. Additional tracheotomy, extubation difficulties, craniotomy, hydrocephalus, increased muscle tone, infection or poor control of underlying disease may impede recovery of consciousness in DoC patients. When rehabilitation physicians, therapists, and care teams work together to overcome these disadvantages, some patients show some progress in their level of consciousness. Therefore, in the process of DoC rehabilitation, it is necessary to reduce as much as possible the factors that are not conducive to the recovery of the patient’s level of consciousness and to reduce the use of drugs that affect the state of consciousness. It is also necessary to create favorable conditions for recovery of consciousness. For example, medication, rehabilitation and acupuncture can be used to speed up the recovery of consciousness.

### 4.4 Safety of rTMS in DoC

When using rTMS as an intervention, rehabilitation physicians and therapists must screen DoC patients for several contraindications. Contraindications include potential effects on brain damage and seizure induction. Some studies have found that rTMS can cause patients to experience mild side effects such as dizziness, headaches, and nausea, but these symptoms quickly resolve when rTMS is stopped (57). Although no reports of dizziness or headache with rTMS were found in the medical records, this minor side effect cannot be ruled out in patients. Because the patient or rehabilitation therapist may not report this to the rehabilitation physician, or because the symptoms are mild, the rehabilitation physician may not pay attention to documenting this status. A study of the risk of using rTMS in patients with DoC following traumatic brain injury found a low rate of rTMS-induced seizures (58). In summary, rTMS can be considered a relatively safe intervention. Therefore, the risk–benefit ratio must be carefully assessed when a patient experiences an adverse reaction.

### 4.5 Study limitations

First, this was a single-center, retrospective study that was affected by sample size and did not include subgroup analyses of ischemic–hypoxic encephalopathy and DoC due to other causes. Second, we only used the GCS to evaluate consciousness levels in this study due to limitations in the medical records. The GCS has difficulty distinguishing between a vegetative state and a minimal conscious state. Additionally, the GCS was insufficiently sensitive to chronic DoC. This may have reduced the level of argumentation for rTMS efficacy and safety evaluations. Third, the degree of brain damage in patients with DoC is variable, and the sample size of this study was insufficient to support too many subgroup analyses. Fourth, a retrospective design essentially lacks random distribution. Despite the use of multifactor correction, the retrospective design cannot eliminate selection bias caused by the joint decision-making of doctors and patients. Patients treated with rTMS may exhibit systematic differences that are difficult to measure, which could introduce bias into the results. In addition, the lack of long-term follow-up data makes it difficult to assess the long-term effects of rTMS treatment.

### 4.6 Implications for future research

Repetitive transcranial magnetic stimulation shows promise as a treatment for disturbances of consciousness. However, the optimal stimulation parameters need to be further optimized and validated. Such as stimulation frequency, stimulation duration and stimulation target area to determine a more appropriate stimulation dose to increase the level of consciousness in patients with DoC. The prognosis of DoC is influenced by a variety of factors, and how to select the best treatment plan for DoC with different levels of injury and etiology is the next direction of research. Future research should focus on refining treatment options. Identifying subgroups of patients most likely to benefit from rTMS by conducting multicenter, large-sample, randomized controlled trials. Explore new ways to improve recovery of consciousness in patients with DoC. In addition, the long-term effects and mechanisms of rTMS treatment need to be further studied and researched. To more accurately assess level of consciousness and better inform clinical decision making, future efficacy assessments based on Coma Recovery Scale-Revised, electroencephalogram, and neuroimaging are needed.

## 5 Conclusion

In conclusion, rTMS did not significantly improve the GCS scores of patients with DoC. However, it may improve the level of consciousness of patients with stroke-induced or moderate DoC. Nevertheless, this conclusion should be interpreted with caution. As this was a retrospective study, medical records were recorded by a specific rehabilitation physician and there was subjectivity, which may have introduced recording bias. In addition, the results do not fully control for potential confounding factors. Therefore, more rigorous and standardized randomized controlled trials are needed to validate the efficacy and safety of rTMS in patients with DoC.

## Data Availability

The original contributions presented in the study are included in the article/supplementary material, further inquiries can be directed to the corresponding author/s.
